# Stathmin! An Immunohistochemical Analysis of the Novel Marker in Oral Squamous Cell Carcinoma and Oral Leukoplakia

**DOI:** 10.31557/APJCP.2020.21.11.3317

**Published:** 2020-11

**Authors:** Purnima Vadla, Sivaranjani Yeluri, G Deepthi, Venkateswara Rao Guttikonda, Sravya Taneeru, Srikanth Naramala

**Affiliations:** *Department of Oral Pathology and Microbiology, Mamata Dental College, Khammam, Telangana, India. *

**Keywords:** Stathmin, oral squamous, cell carcinoma, oral epithelial dysplasia, biomarker, poor prognosis

## Abstract

**Background:**

Stathmin is an intracellular phosphoprotein that controls the microtubule dynamics by further regulating proper attachment and alignment of chromosomes in a dividing cell. Thus, any mutation or aberrantly expressed protein that reduces the fidelity of spindle assembly will enhance chromosomal instability contributing to aneuploidy. Oral Squamous Cell Carcinoma is an extensively studied malignancy that occurs due to accumulated genetic changes due to carcinogens. The current study is done to evaluate the stathmin role and its expression in OSCC and Oral epithelial dysplasia (OED).

**Objective:**

The aim of the present study is to evaluate the role of stathmin in OSCC and Oral dysplasia and also to correlate the expression of Stathmin with respect to the different histopathological grades of OED and OSCC.

**Materials and Methods:**

30 neutral buffered formalin fixed, paraffin embedded (FFPE) tissues of Oral Leukoplakia/OED and 30 FFPE tissues of OSCC were subjected to immunohistochemistry with stathmin antibody. Five fields of each case with 300 cells were examined and a mean percentage of positive–stained slides were determined. The percentages were recorded accordingly with their respective histological grades. The results were analysed statistically.

**Results:**

The results of the present study demonstrated higher mean values of stathmin in tissues with OSCC (2.50) compared to leukoplakia (2.11) and normal tissues (0.00) with a high level of statistical significance (0.0001). There is also an increase in the percentage levels of stathmin with increase in the histological grade of differentiation in OSCC as well as leukoplakia.

**Conclusion:**

The present study found a statistical correlation between increased grades of the disease with expression levels of stathmin. This confirms that stathmin expression can contribute to disease progression and that stathmin might have a potential role as an early diagnostic biomarker and can be a therapeutic target for OSCC.

## Introduction

Cell division involves segregation of duplicated chromosomes by the bipolar spindle, which is made up of heterodimer polymers of tubulin termed microtubules (MT) (Belmont et al., 1996). The spindle MTs are the molecular machines used to segregate chromosomes to the daughter cells using kinetochores. They are the proteinaceous structures that assembles on the centromeric DNA and plays several roles during mitosis (Biggins et al., 2003). Firstly, they are the site of attachment of chromosome to microtubules, which allows for the proper alignment and segregation of chromosomes. Kinetochores also contain motor proteins that help in complex movements of chromosomes during mitosis. Lastly, it serves as an assembly of checkpoint machinery which assures proper attachment and alignment of chromosomes prior to initiation of anaphase. If any errors found in the attachment of MTs, this mitotic spindle checkpoint will act as a signal transduction mechanism which causes cell cycle arrest (Biggins et al., 2003).

Stathmin, also called oncoprotein 18, firstly identified in neuroendocrine cells, plays a critical role during signal transduction in modulation and control of microtubule polymerization dynamics (Nemunaitis et al., 2012; Tian et al., 2013). This dynamics was best described as an alternating pattern of stabilization and destabilization. Reactivation of the phosphorylated stathmin to dephosphosphorylated state has to be done before cells exit mitosis. Any interference in stathmin function results in reduced cellular proliferation and accumulation of cells in the G2/M phases of the cell cycle. Forced expression leads to abnormalities in mitotic spindle assembly and arrest of cells in the early stages of mitosis (Rubin et al., 2004). Thus, a tightly regulated sequenced pattern of STMN1 phosphorylation and de-phosphorylation is necessary for entry into prophase and, terminally, into cytokinesis, respectively (Nemunaitis et al., 2012).

Defects in the mitotic checkpoint result in chromosomal instability and are manifested as aneuploidy. Most malignant tumours show various degrees of chromosomal instability, which are caused by many different types of defects that promote spindle aberrations, such as impairment of the mitotic checkpoint response, aberrant number of spindle poles, spindle attachment defects, and defects in chromosome cohesion/ disjunction. Thus, any type of mutation or aberrantly expressed protein that reduces the fidelity of spindle assembly will enhance chromosomal instability and can thus be considered to be aneugenic since such defects contribute to aneuploidy (Rubin et al., 2004).

Numerous studies had reported that stathmin was overexpressed with poor prognosis and chemoresistance in a variety of human malignancies like acute leukemia, prostate, gastric, breast and ovarian cancer (Luo et al., 1994; Friedrich et al., 1995; Alaiya et al., 1997; Curmi et al., 2000; Akhtar et al., 2013). Head-and-neck cancers constitute the epithelial malignancies that arise from paranasal sinuses, nasal cavity, oral cavity, pharynx and larynx. Oral squamous cell carcinoma (OSCC) being the commonest of head-and-neck cancers, accounts for 354,864 new cases and 177,384 cancer-related deaths worldwide in 2018 (Deepthi et al., 2020). 

Pathogenesis of OSCC results from the combination of multiple molecular events that develop from both, an individual’s genetic predisposition and exposure to carcinogens that could damage individual genes. These accumulated genetic changes leads to causation of OSCC and in some instances via a clinically evident oral potentially malignant disorder (OPMD), which may undergo sequential pathological changes from dysplasia to invasive carcinoma (Choi et al., 2008). Oral leukoplakia (OL) is the most common of OPMDs, with a risk of malignant transformation ranging between 0.13 to 34%, which was revealed in a systemic review of 24 studies (Warnakulasuriya et al., 2016). Despite a considerable progress in understanding the pathogenesis of OSCC, the treatment strategy has been quite disorganised with respect to advanced stage OSCCs; therefore, it’s quite important to find a biomarker to differentiate an early stage OSCC from a late stage one. 

However, the overexpression and prognostic impact of stathmin in other poorly differentiated malignancies have been elucidated, but it is not quite clear of the role of stathmin in different grades of OSCC as well as in OPMDs. Taking this objective into account, the present study was undertaken to compare and evaluate the expression of Stathmin in different histological grades of Oral Leukoplakia (OL) and Oral Squamous cell Carcinoma (OSCC) using immunohistochemistry. 

## Materials and Methods

The content and purpose of this retrospective study has been approved by the Institutional ethical committee with IEC number MDC_T_D158803021. 30 neutral buffered formalin fixed, paraffin embedded (FFPE) tissues of Oral Leukoplakia and 30 FFPE tissues of OSCC were retrieved from the Department of Oral Pathology and Microbiology, Mamata Dental College, Khammam for the purpose of this study and compared with that of normal tissues. Hematoxylin and eosin (HandE) stained preparations of each case were reviewed and histologically graded OL using World Health Organization criteria and cases of OSCC were graded histopathologically using Modified Broder’s grading system. The identified slides were grouped into categories and were illustrated in Table 1. 

2-3 serial sections of 3μm thickness were made from FFPE tissues and taken onto silanized slides. The sections were deparaffinised by keeping the slides on the slide warmer at 60º C for 15-20 min followed by rehydration of the sections through 2 changes of xylene, and then into absolute alcohol, 95% alcohol, 70% alcohol for 5 min respectively. Then the slides were kept immersed in distilled water for 30 seconds. Antigen retrieval was done by placing the slides in a plastic container containing a metal slide rack which in turn was kept in a microwave oven containing boiling Tris buffered saline (pH 9.0 - 9.2). The slides were heated four times at 100ºC for 5 minutes and then allowed to cool down to room temperature. 

*Immunohistochemistry staining procedure*

All the reagents stored in the refrigerator were brought to room temperature (24º-28º C) prior to immunostaining. All the incubations were performed at room temperature using a humidifying chamber. The sections were washed gently in Phosphate Buffered Saline (PBS) for three times for 2 mins each. Endogenous peroxidase activity was quenched by placing the slides in 0.3% H_2_O_2_ for 15-20 minutes followed by gentle washing with PBS three times for 2 min each. After tapping off the excess buffer from the slide, the sections were covered with Power Block (Contains casein and proprietary additives in phosphate buffered saline with 15mM sodium azide) for 15-20 minutes. Primary antibody application was done after Power Block was tapped off; the sections were covered completely with pre-diluted Stathmin primary antibody except the negative control (Non- immune serum in Phosphate Buffered Saline with 0.09% sodium azide). The slides were incubated for 1 hour at 21ºC in a humidifying chamber followed by gentle washing with PBS three times for 2 min each. Super Enhancer is applied later and left for 30 minutes and then washed again with PBS three times for 2 min each. 

A MultiLink secondary antibody (Pre-diluted biotinylated anti-immunoglobulins in phosphate buffered saline with carrier protein and 0.09% sodium azide) application was done after tapping off the excess buffer, the sections were then incubated with secondary antibody for 30 minutes. The slides were then washed gently with PBS three times for 2 min each. Excess buffer was tapped off and tissue sections were completely covered with freshly prepared substrate chromogen solution (1 ml DAB buffer with 2 drops DAB chromogen) using Pasteur pipette for 10 minutes followed by washing gently with distilled water for 2 minutes. Counterstaining was done by immersing the slides in Mayer’s hematoxylin for 2 minutes, then washed gently under running tap water for bluing. Dehydration by taking the tissue sections through absolute alcohol, 95% alcohol, 70% alcohol for 5 min respectively. The sections were kept immersed in xylene bath and later were mounted using DPX (Lendrum Di-n-butyl Phthalate in Xylene).

**Figure 1:**
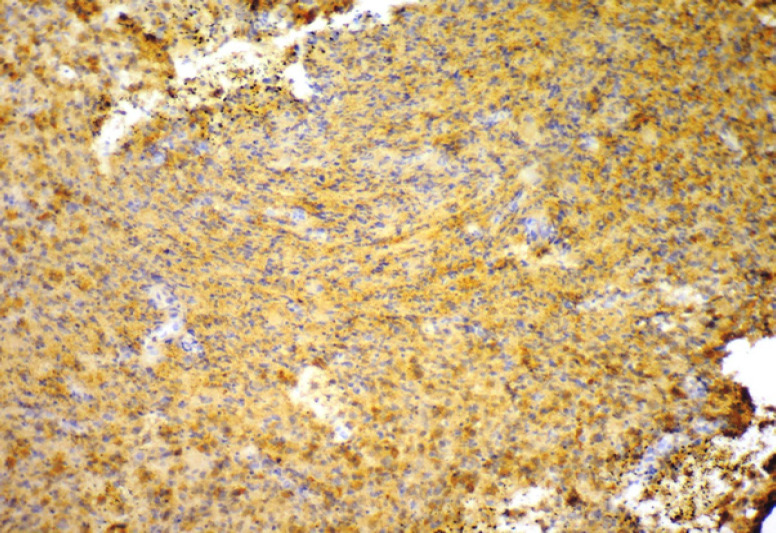
Photomicrograph of Brain as a Positive Control for Stathmin Immunoexpression (10X)

**Figure 2:**
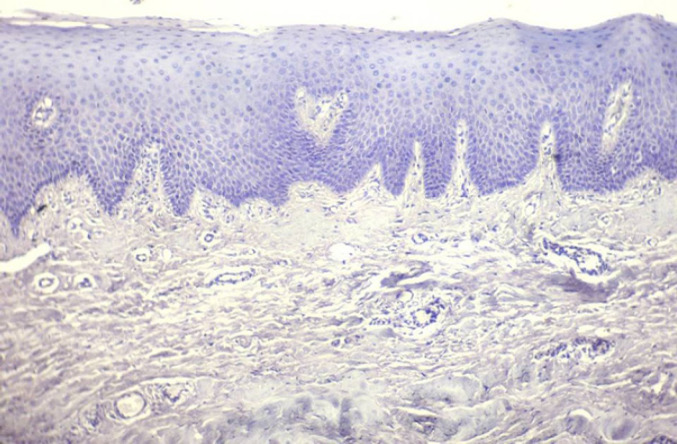
Photomicrograph of Normal Oral Mucosa as a Negative Control for Stathmin Immunoexpression (10X)

Presence of brown coloured end product at the site of target antigen was indicative of positive immunoreactivity. The negative control tissue (Normal mucosal tissue omitting the primary antibody) demonstrated absence of staining. Brain tissue was taken as positive control (Figure 1) with each batch of staining and normal oral mucosal tissue was taken as negative control (Figure 2). The evaluation of study cases was done subsequently in a similar way and was graded as positive or negative. To enumerate the Stathmin stained slides, 300 cells were examined manually in at least 5 areas and a mean percentage of positive–stained slides were determined. Then, each sample was assigned to one of the following staining scores: 0 – Less than 10%, 1 – 11 to 25%, 2 – 26 to 50%, 3 – 51 to 75%, 4 – 76 to 90% and 5 – 91 to 100% (Fedchenko et al., 2014).The slides were examined by two observers independently to prevent interobserver bias. Intraclass correlation coefficient (ICC) analysis reveals a good agreement between two observers (ICC = 0.9). The results were calculated in percentages and analysed statistically through ANOVA test analysis using Social Sciences software, version 20.0 (IBM Corp. Released 2011, IBM SPSS Statistics for windows version 20.0, Armonk, NY: USA). p-value of 0.0001 was considered statistically significant. 

## Results

30 tissues of Oral leukoplakia (n=30) and Oral squamous cell carcinoma (n=30) each were evaluated for the immuohistochemical expression of Stathmin and compared with normal tissues (n=30). 

**Graph 1:**
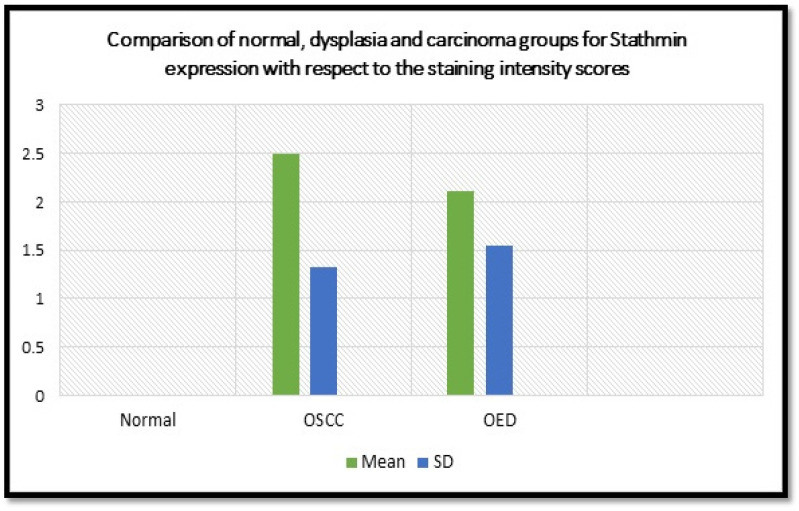
Graphical Representation of Comparison of Normal, OED and OSCC Groups for Stathmin Expression with Respect to the Staining Intensity Scores

In the dysplasia (OL) group, of 30 cases, the staining score was found to be 0 in three cases (10%), 1 in fifteen cases (50%), 2 in six cases (20%), 3 in two cases (6.66%), 4 in four cases (13.33%). Out of 30 cases in OSCC group, the staining score was found to be 0 in one case (3.33%), 1 in seven cases (23.33%), 2 in nine cases (30%), 3 in seven cases (23.33%), 4 in two cases (6.66%), 5 in four cases (13.33%). In the normal group, the staining score was found to 0 in thirty cases (100%). When comparison was made with respect to staining scores between normal, dysplasia and carcinoma groups, the results were found to be statistically significant with a p value of 0.0001 (Table 2, Graph 1).

**Figure 3:**
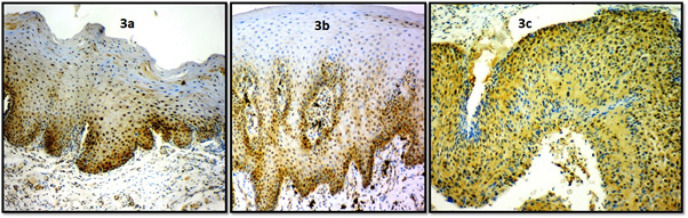
Photomicrograph of Stathmin Immunoexpression in 3a, Mild dysplasia (10X); 3b, Moderate dysplasia (10X); 3c, Severe dysplasia (10X)

Analyses among the dysplasia cases show that, out of 16 mild dysplasia cases, the staining score was found to be 0 in three cases (18.75%), 1 in thirteen cases (81.25%). In 10 moderate dysplasia cases, the staining score was found to be 1 in two cases (20%), 2 in six cases (60%), 3 in two cases (20%). In 4 severe dysplasia cases, the staining score was found to be 4 in four cases (100%) (Figure 3a, 3b and 3c). A statistically significant difference was observed between various histopathological grades of dysplasia with respect to immunohistochemistry scores with a p value of 0.0001 (Table 3)

**Figure 4:**
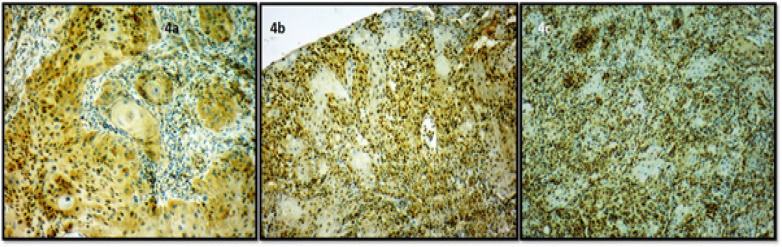
Photomicrograph of Stathmin Immunoexpression in 4a, Well differentiated OSCC (10X); 4b, Moderately differentiated OSCC (10X); 4c, Poorly differentiated OSCC (10X).

Out of 14 well differentiated cases of OSCC, the staining score was found to be 0 in one case (7.14%), 1 in seven cases (50%), 2 in six cases (42.85%). In 10 moderately differentiated cases, the staining score was 2 in three cases (30%), 3 in seven cases (70%). In 6 poorly differentiated cases, the staining score was found to be 4 in two cases (33.33%), 5 in four cases (66.66) (Figure 4a, 4b and 4c ). A statistically significant difference was observed between various histological grades of OSCC with respect to immunohistochemistry scores with a p-value of 0.0001 (Table 4).

**Table 1:** Distribution of Oral Leukoplakia and OSCC Cases According to Histological Ggrade

	Histological Grade	No. of cases
Oral leukoplakia (n=30)	Mild dysplasia	16
	Moderate dysplasia	10
	Severe dysplasia	4
OSCC (n=30)	Well differentiated OSCC	14
	Moderately differentiated OSCC	10
	Poorly differentiated OSCC	6

**Table 2:** Comparison of Normal, Dysplasia and Carcinoma Groups for Stathmin Expression with Respect to the Staining Intensity Scores Using ANOVA Test

Groups	Mean ± SD	*P*-value
OSCC group	2.50 ±1.33	0.0001*
Dysplasia (OED) group	2.11 ± 1.54	
Normal group	0	

p= 0.0001, *, Significant; SD, standard deviation

**Table 3:** Comparison of Various Histological Grades of Dysplasia (Mild, Moderate, Severe) for Stathmin Expression with Respect to Staining Intensity Scores

Histopathological grading	Mean ± SD	*P*-value
Mild dysplasia	0.55 ± 0.33	0.0001*
Moderate dysplasia	1.97 ± 0.45	0.0001*
Severe dysplasia	3.89 ± 0.52	0.0001*

Kruskal Wallis ANOVA test; p=0.0001; *, Significant; SD, standard deviation

**Table 4:** Comparison of Various Histological Grades of OSCC (Well, Moderate and Poor) for Stathmin Expression with Respect to Staining Intensity Scores

Histopathological grading	Mean ± SD	*P*-value
Well differentiated OSCC	1.38 ± 0.91	0.0001*
Moderately differentiated OSCC	2.98 ± 0.56	0.0001*
Poorly differentiated OSCC	4.57 ± 0.54	0.0001*

Kruskal Wallis ANOVA test; p, 0.0001; *, Significant; SD, standard deviation

## Discussion

The name stathmin is derived from the term ‘stathmos’, the Greek word for ‘relay’ (Nemunaitis et al., 2012). Stathmin plays a critically important role in the regulation of the microtubule cytoskeleton. It controls the microtubule dynamics by promoting depolymerization of microtubules and/or preventing polymerization of tubulin heterodimers. Depolymerisation of microtubules is done either by sequestering free tubulin dimers or by directly inducing microtubule-catastrophe. Various researches on stathmin showed that it is frequently overexpressed in a range of human cancers and also has a close association with cancer cell differentiation, TNM classification and Lymph node metastases (Liu et al., 2013). Overexpression of stathmin maintains proliferation of cancer cell suggesting the role of stathmin in tumorigenesis and tumor development and provides an attractive oncobiological marker and molecular target for cancer therapy (Karst et al., 2011). 

In the present study, it was observed that there was statistically significant increased expression of stathmin in OSCC group (2.50 ±1.33) compared to the OL (dysplasia) group (2.11 ± 1.54) and the normal tissue (0.00). To the best of our knowledge, this study is the first of its kind to compare the expression of stathmin in normal, dysplasia and carcinoma groups. The results also showed a statistically significant increased expression of stathmin from mild dysplasia (0.55 ± 0.33) to severe dysplasia (3.89 ± 0.52). In the intragroup comparison of different grades of OSCC, there was a statistically significant increase from well differentiated OSCC (1.38 ± 0.91) to poorly differentiated OSCC (4.67 ± 0.54). From the observations of previous studies, over expression of Stathmin has been observed in various human cancer cell lines and Stathmin gene plays an important role in mitosis and other cellular processes that could promote cancer growth (Rubin et al., 2004). Hanash et al., (1988) reported that stathmin was found to be highly expressed in primary acute leukemia and lymphoma samples, and leukemia cell lines. It was also reported that stathmin expression was markedly increased when normal lymphocytes are induced to proliferate on mitogenic stimuli. In chronic myelogenous leukemia, stathmin expression was shown to significantly increase when the disease progresses into the more proliferative stage.

Marafioti et al., (2013) in their study on immunohistochemical and molecular analyses of a large series of follicular lymphomas suggested that stathmin could be used as a novel immunohistochemical and a diagnostic marker. They also suggested that the increased expression of Stathmin on the other hand could be correlated with the higher grade of the tumour. Curmi et al., (2000) observed stathmin overexpression in breast carcinomas which was correlated with higher histological grade, advance pathological state, tumour recurrence and disease progression. Wei et al., 92008) observed stathmin expression in all ovarian cancer samples and higher levels were observed in the metastatic tumours. They also found overexpression of Stathmin in higher grades of serous ovarian carcinomas. Trovik et al., (2011) found overexpression of Stathmin in endometrial cancer patients and correlated the expression with aneuploidy and high grade of the disease. They also suggested that it could be predictor for metastasis. Lu et al., (2014) reported that Stathmin was overexpressed in pancreatic cancer samples and high Stathmin levels were correlated with tumour size, reduced cellular proliferation, clonogenicity, cell cycle arrest and negatively impacted the overall survival rate. The results of the present study were in accordance with. Kang et al., (2012) reported that high Stathmin expression in the gastric carcinoma cell lines and predicted poor prognosis of the same as the rate of expression increases and also subsequently decreases the overall survival rate which was in accordance with the present study. All the above mentioned were in concordance with the results of the present study that stathmin expression levels were higher with increased grade of the tumour. 

Amongst the head and neck cancers, studies reported that Stathmin was expressed immunohistochemically in OSCC, nasopharyngeal carcinoma and adenoid cystic carcinoma (Kouzu et al., 2000; Nakashima et al., 2006; Cheng et al., 2008; Hsu et al., 2014). Cheng et al., (2008) identified increased expression of stathmin in primary nasopharyngeal carcinoma and its expression was associated with the advanced stages of the disease. This was also associated with higher grade of the tumour and was a predictor of the worst prognosis of the disease. Nakashima et al., (2006) reported that they found an increased expression of stathmin in adenoid cystic carcinoma which was demonstrated using Using 2- dimensional differential in-gel electrophoresis. Kouzu et al., (2000) in their study found that there wasa significant correlation of stathmin with clinical staging of OSCC. Upon immunohistochemistry analysis, 65% of the OSCCs were positive for stathmin, and no immunoreaction was observed in corresponding normal tissues. Real-time quantitative reverse transcriptase–polymerase chain reaction data were found consistent with the protein expression status. Furthermore, they found a statistical correlation between the protein expression status and disease-free survival suggesting that the expression of stathmin could contribute to cancer progression/prognosis, and that stathmin may have potential as a biomarker and a therapeutic target for OSCC.

More direct evidence for a role of Op18/ Stathmin in tumorigenesis is from identification of the somatic Q183E mutation in one allele from a human adenocarcinoma biopsy. This allele was shown to confer transforming properties on the mutated protein expressed in murine NIH-3T3 cells. The somatic Q183E mutation is the only mutation of Op18 identified so far, and it has only been observed in a single tumor. Expression of the mutant Op18-Q18E protein in NIH-3T3 cells has been found to facilitate tumor growth in immune-deficient mice as well as increasing the basal frequency of focus formation in soft agar. Together, these observations provide a strong case for the significance of this mutation in the original tumor (Cheng et al., 2008).

Osone et al., (2019) evaluated the expression of stathmin in colorectal dysplasia and cancer in patients with Ulcerative colitis, where they found, expression of stathmin was detected in 95.7% of sections of dysplasia and cancerous lesions and its expression was not detected in any section of noncancerous lesions. With these observations, they suggested that stathmin expression might be more accurate and useful than the current diagnostic marker, p53, for the diagnosis of dysplasia and cancer in patients with ulcerative colitis and further suggested that stathmin expression in the colonic mucosa of patients with ulcerative colitis might be useful as an early diagnostic marker of dysplasia and colitic cancer. This goes well in accordance with the present study which evaluated stathmin expression in oral dysplasia and OSCC. This study presents some limitations with respect to sample size, where the efficiency of these markers can be estimated using well designed, multi-institutional trial with larger sample size. 

In conclusion, a comparison of expression of stathmin was observed with respect to OSCC, Oral dysplasia and normal tissues. Furthermore, we found a statistical correlation between increased grades of the disease with expression levels of stathmin. These results suggest that stathmin expression contribute to disease progression and that stathmin might have a potential role as a early diagnostic biomarker and can be a therapeutic target for OSCC. 
